# Role of Semaphorins in Ischemic Stroke

**DOI:** 10.3389/fnmol.2022.848506

**Published:** 2022-03-08

**Authors:** Huaping Du, Yuan Xu, Li Zhu

**Affiliations:** ^1^Department of Neurology, Suzhou Ninth Hospital Affiliated to Soochow University, Suzhou, China; ^2^Suzhou Key Laboratory of Thrombosis and Vascular Biology, Jiangsu Key Laboratory of Preventive and Translational Medicine for Geriatric Diseases, Collaborative Innovation Center of Hematology of Jiangsu Province, National Clinical Research Center for Hematologic Diseases, Cyrus Tang Medical Institute, Soochow University, Suzhou, China

**Keywords:** semaphorins, ischemic stroke, neurovascular unit, neurons, glial cells, vasculature

## Abstract

Ischemic stroke is one of the major causes of neurological morbidity and mortality in the world. Although the management of ischemic stroke has been improved significantly, it still imposes a huge burden on the health and property. The integrity of the neurovascular unit (NVU) is closely related with the prognosis of ischemic stroke. Growing evidence has shown that semaphorins, a family of axon guidance cues, play a pivotal role in multiple pathophysiological processes in NVU after ischemia, such as regulating the immune system, angiogenesis, and neuroprotection. Modulating the NVU function *via* semaphorin signaling has a potential to develop a novel therapeutic strategy for ischemic stroke. We, therefore, review recent progresses on the role of semphorin family members in neurons, glial cells and vasculature after ischemic stroke.

## Introduction

Ischemic stroke is one of the leading causes resulting in high mortality and substantial loss of neurological function in the world (Tsai et al., 2013). Ischemic stroke occurs due to disruption or significant reduction in the blood flow to a brain region, resulting in permanent neurological deficits or death. Relative to weight, the brain oxygen consumption is very high (accounts for 20–30% of the total oxygen consumption) and needs more ATP through mitochondrial electron transport chain to maintain cell viability (Dienel and Hertz, 2001; Lin and Powers, 2018). Brain has no energy reserve, and aerobic glycolysis is the brain’s principal source of ATP (Cunnane et al., 2020). Therefore, brain is more susceptible to hypoxia. Pathogenic mechanisms following ischemic stroke including excitotoxicity, oxidative stress, inflammation, and apoptosis (Datta et al., 2020). Previous studies highlighted ischemia-induced neuronal damage and neuronal protection has been emphasized during treatment (Wang et al., 2021). Current studies focus on the role of the neurovascular unit (NVU) in the pathophysiological processes of ischemic stroke (Steliga et al., 2020). Regulation of the NVU in multiple ways promotes the rehabilitation of neurological function, such as maintaining blood-brain barrier (BBB) integrity and regulating glial cell activity. A number of studies confirmed that semaphorins affect the prognosis of ischemic stroke by regulating NVU (Wei et al., 2015; Hira et al., 2018; Zhou et al., 2018b; Zhao et al., 2021). Increased evidence indicated that semaphorins regulate cell morphology and physiological function during the development of cardiovascular, immune, endocrine, respiratory and central nervous systems (CNS) (Carulli et al., 2021). Moreover, semaphorins play an important role in the pathological processes of the diseases in these organ systems. In CNS, semaphorins have been shown to be involved in many diseases, and several semaphorin members have been reported to participate in pathogenic process of ischemic stroke (Sawano et al., 2015; You et al., 2019; MacKeigan et al., 2020). These evidences point to a role of semaphorins in the regulation of ischemic stroke. Therefore, semaphorins are considered as a promising therapeutic tool in ischemic stroke. In this review, we focus on the role of semaphorins in NVU after ischemic stroke.

## The Neurovascular Unit and Stroke

Risk factors for ischemic stroke includes age, hypertension, diabetes, atrial fibrillation, hypercholesterolemia, etc. (Tsai et al., 2013; Sarikaya et al., 2015). No matter the precipitating event, the result of ischemic stroke is cerebral cell lacking oxygen and energy, leading to disturbed cellular metabolism until death at the molecular level (Sekerdag et al., 2018). The mechanism for the brain injury caused by ischemia includes excitotoxicity, oxidative, and nitrative stress, inflammation and apoptosis (Khoshnam et al., 2017). Multiple types of cells, including neurons, glial cells, endothelial cells and pericytes, undergo those pathophysiological process and lead to cell destruction finally (Hou and MacManus, 2002). Once ischemia occurs, cells especially neurons are unable to sustain their normal function due to hypoxia. Then, ischemic brain tissue can release inflammatory cytokines, increase oxygen radical and excitatory neurotransmitter production, and disrupt the BBB, which causes further tissue damage (Khoshnam et al., 2017; Jiang et al., 2018). Inhibition of those pathophysiological process can mitigate cell damage (Tao et al., 2020). NVU dysfunction directly promotes the breakdown of the BBB, and present theory emphasizes that NVU repair is important to improve functional recovery, namely neurorepair (Davis et al., 2021; Wang et al., 2021).

**FIGURE 1:**
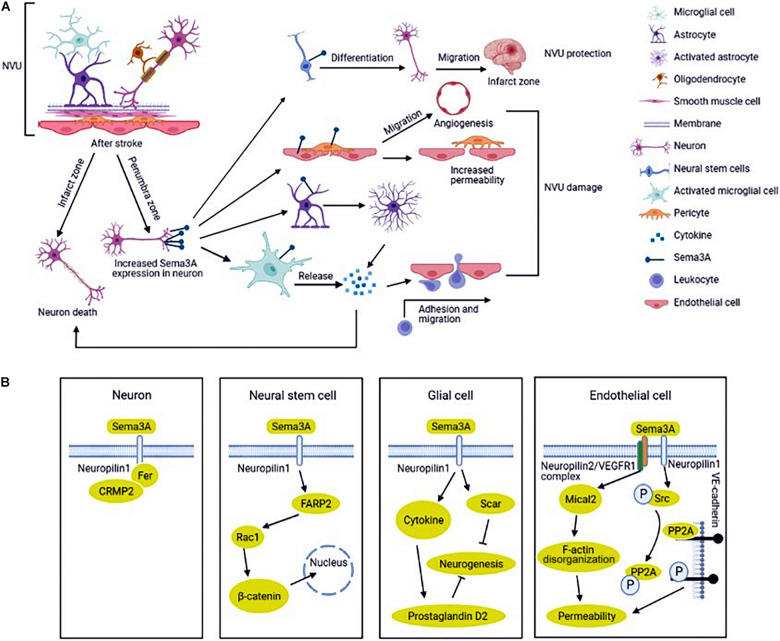
Regulation and mechanism of semaphorin 3A (Sema3A) in neurovascular unit (NVU) after ischemic stroke. **(A)** NVU is consisted of neurons, glial cells, endothelial cells, smooth muscle cells (SMCs), pericytes, and extracellular matrix. The expression of Sema3A in NVU is up-regulated in ischemic stroke. Sema3A inhibits the NVU function by inducing neuron death, activating the inflammatory response, reducing the function of endothelial cells and pericytes to increase vascular permeability, and increasing neovascularization. In addition, Sema3A also promotes the differentiation of neural stem cells (NSCs) into neural cells in the injured cortical tissue. **(B)** Sema3A is upregulated in ischemic stroke, which recruits Fer and CRMP2 (Collapsin response mediator protein) binding to its receptor NRP1(neuropilin), inducing neuron death. The cross-talk between Sema3A and Wnt/β-catenin pathways participates in the regeneration process. Sema3A inhibit axonal growth *via* increasing inprostaglandin D2 synthase expression and glial scar information in glial cells. Sema3A bind to the NRP2 (neuropilin)/VEGFR1 (vascular endothelial growth factor receptor) receptor complex and disrupt PP2A (protein phosphatase 2A) interaction with VE (Vascular endothelial)-cadherin, increasing vascular permeability in endothelial cells.

NVU is consisted of neurons, glial cells, endothelial cells, smooth muscle cells (SMCs), pericytes, and extracellular matrix (Figure 1A; Iadecola, 2017). Neurons, the core of the NVU, detect very little changes of nutrients and oxygen, transmit associated signals to other cells (Banerjee and Bhat, 2007). Glial cells exert pivotal effects during ischemic stroke. Microglial cells are rapidly activated after ischemic stroke and release inflammatory cytokines which activate astrocytes. Astrocytes, by secreting proinflammatory cytokines, chemokines, and matrix metalloproteinase 9, communicate simultaneously with both neurons and blood vessels and then trigger the remodeling of NVU (Gordon et al., 2008; Lopez-Bayghen and Ortega, 2011). The functional characteristics of astrocytes are altered at the different stages of ischemic stroke. Astrocytes limit brain damage in the acute stages and inhibit axon regeneration in the chronic stages (Xu et al., 2020). Different polarizations of astrocytes also have different functional characteristics under pathological conditions (Liu et al., 2020). Endothelial cells produce vascular active factors to control vascular tone, maintain vascular permeability and integrity of NVU together with SMCs and pericytes (Duchemin et al., 2012). Cross talk between cells in NVU through a complex and delicate network. Integrity of NVU is highly important to maintain the homeostasis of brain microenvironment and regulate cerebral blood flow (Armstead and Raghupathi, 2011).

Brain ischemic injury can stimulate the NVU to activate inflammatory cells, upregulate adhesion molecules, release multiple cytokines such as interleukins-1β (IL-1β) and tumor necrosis factor-α (TNF-α) (Wang et al., 2021). Inflammatory factor exacerbates cellular damage and death. Inhibition of inflammatory response can significantly improve the prognosis of stroke. In addition, astrocytes, one of the most important components of NVU, can secrete neurotrophic factors that guide neuronal migration and facilitate neuronal and axonal regeneration (Xu et al., 2020). Therefore, in-depth study of the NVU provides potential target for ischemic stroke treatment.

## Role of Semaphorins in Ischemic Stroke

**FIGURE 2:**
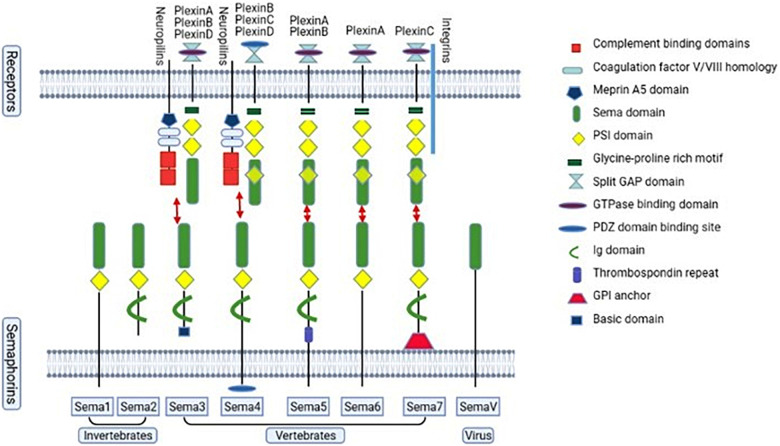
The semaphorin family and the vertebrate semaphorins’ main receptors. The semaphorin family proteins are divided into eight classes, class 1–7 and class V. Classes 1–2 and class 5C are found in invertebrates, while classes 3–7 are found in vertebrates. Class V is found in virus. Classes 1–7 consist of a large sema domain and a plexin-sema-integrin (PSI) domain. Classes 2–4 and class 7 contain an immunoglobulin (Ig)-like domain. Class 3 contains a basic domain. Class 4 contains a PDZ (Post-synaptic density-95, disks-large and zonula occludens-1) binding site. Class 5 contains a thrombospondin repeat. Class 7 is glycosylphosphatidylinositol (GPI)-linked. Neurophilins and plexins are semaphorin 3 receptors. For semaphorin 4, the main receptors are neurophilins and plexinB, C, D. For semaphorin 5, the main receptors are plexinA and plexinB. PlexinA are the main receptors of semaphorin 6. PlexinC and integrin are the main receptors for semaphorin 7.

Semaphorins are a large family of axon guidance cues, which consist of a sema domain (a specific region of about 500 amino acids), a plexin-semaphorin-integrin (PSI) domain, and distinct protein domains (Figure 2; Kolodkin et al., 1993; Lu and Zhu, 2020). Based on the structure and distribution characteristics, semaphorin family proteins are divided into eight classes (Hu and Zhu, 2018; Limoni, 2021). Class 1–2 and class 5C are found in invertebrates, while classes 3–7 are found in vertebrates and class V is found in virus. In vertebrates, semaphorin 3 and 4 have 7 members, namely A–G; semaphorin 5 has 2 members, named 5A and 5B; semaphorin 6 has 4 members, named 6A to 6D; semaphorin 7 has only one member (Hu and Zhu, 2018). In addition, class 1, 4, 5, and 6 are bound to the cell membrane through a transmembrane domain; class 2, 3, and V can be secreted; and class 7 is the only glycosylphosphatidylinositol (GPI)-anchored protein. The structure of semaphorins is shown in Figure 2. The functions of semaphorins are mediated by their receptors plexins and neuropilins (Figure 2; Raper, 2000; Nissen and Tsirka, 2016; Junqueira et al., 2021). Invertebrates have plexins A and B, and vertebrates have plexins A to D. However, class 3 mediated signaling requires the binding of both plexins and neuropilins (Hu and Zhu, 2018). Moreover, other molecules, such as Otk (transmembrane protein Off-track) and CD27, work as a part of receptor complex of the semaphorins or directly as their receptors (Winberg et al., 2001; Xue et al., 2016).

Early studies in neurons revealed that semaphorins play attractive or repulsive role in axonal growth, regulating the precise wiring of neural architecture. During the last three decades, semaphorins have been considered as key regulators of cell physiological process in different organ systems, especially in the nervous system, the circulatory system, and the immune system (Carulli et al., 2021). The major function of semaphorins is to modulate cytoskeleton motion and cell adhesion, and thereby affect cell morphology, growth, differentiation, migration and survival (Nakamura et al., 2000). In addition, a great deal of progresses has been made in defining the roles of semaphorins in the regulation of CNS diseases under pathological conditions, such as ischemic stroke. Emerging evidence suggests that semaphorins are involved in the development of atherosclerosis and thrombus formation (Zhu et al., 2007; Hu and Zhu, 2018). Semaphorins act as important regulators of neurogenesis, cell migration, cell apoptosis, vascular morphogenesis, angiogenesis and immune responses in the pathologic process of ischemic stroke (Tian et al., 2009; Limoni and Niquille, 2021; Yu et al., 2021). Semaphorin 3A (Sema3A) and semaphorin 4D (Sema4D) are important for cell apoptosis, inflammatory response, neurogenesis and angiogenesis. Semaphorin 3B (Sema3B) can affect the integrity of neuron dendritic structure. Semaphorin 3E (Sema3E) suppresses the migration capacity of pericytes toward endothelial cells, increases the vascular permeability, and damnifies NVU. Semaphorin 4B (Sema4B) serves as an astrocyte receptor to regulate astrogliosis after ischemic stroke. Semaphorin 6B (Sema6B) and semaphorin 7A (Sema7A) mainly involves in angiogenesis and vascular permeability.

## Semaphorin 3A

Sema3A was first found in the chicken brain and induced the collapse and paralysis of neuronal growth cones (Kolodkin et al., 1993; Luo et al., 1993). It is the prototypical and deeply understood member of the semaphorin family. Sema3A and its receptors (neuropilins and plexins) were found to express in the nervous system including neurons, microglial cells, astrocytes, endothelial cells and oligodendrocytes (Takahashi et al., 1999; Fujita et al., 2001; Hashimoto et al., 2004). Sema3A binds to its high affinity receptor neuropilins, but the signal cannot be transmitted effectively. The signal delivery process simultaneously requires another receptor plexins to form complex. The complex is responsible for initiating the signal transduction and leading to growth cone collapse and axon repulsion (Nakamura et al., 2000). Sema3A is closely associated with ischemic stroke and affects stroke recovery (Pekcec et al., 2013). Oxygen-glucose deprivation (OGD) is widely used as an *in vitro* model for stroke, showing similarities with the *in vivo* models of brain ischemia (Tasca et al., 2015). The expressions of Sema3A and neuropilin (NRP) -1 in cultured rat cortical neurons are up-regulated after OGD treatment, which in turn take part in the neuron apoptosis (He et al., 2018; Yang et al., 2019). In the middle cerebral artery occlusion (MCAO) model, Fujita et al. (2001) found that the expression of Sema3A and neuropilins is temporally upregulated and could not induce neuron death in the non-infarcted parietal cortex on the lesion side. However, up-regulated Sema3A and its receptors, lasting for a longer time, could activate glial cells to induce neuronal death in infarct lesion (Figure 1A; Fujita et al., 2001). Hypoxia increases oxygen radical production in neuronal cells (Khoshnam et al., 2017). Regulating Sema3A expression can decrease OGD-mediated cell damage by reducing neuronal oxidative stress and apoptosis (Yang et al., 2021; Zhao et al., 2021). A number of studies have indicated that the mechanism involved in cerebral ischemia-induced neuronal death and neurovascular unit damage is NRP1 (not neuropilin-2, NRP2)/Fer/CRMPs (Collapsin response mediator protein) pathway (Figure 1B; Aylsworth et al., 2009; Hou et al., 2009, 2010; Jiang et al., 2010; Whitehead et al., 2010). CRMP has been identified as an intracellular signaling mediator for Sema3A (Makihara et al., 2016). In this signaling, cyclin-dependent kinase 5 (Cdk5) primarily phosphorylates the residues of Ser522 of CRMP2. Glycogen synthase kinase-3β(GSK-3β) subsequently phosphorylates the residues of Thr509 and Thr514 of CRMP2 (Nakamura et al., 2018). Another study indicated that the nuclear transcription factor E2F1 plays an important role in modulating neuronal death in response to cerebral ischemia by enhancing the NRP1 level *via* binding NRP1 promoter sequence (Jiang et al., 2007). Nonetheless, Beck et al. (2002) showed that Sema3A, 3C, and 3F appeared to be strongly downregulated in the infarcted and peri-infarct cortical neurons. The authors speculated that low level of Sema3 in neurons could promoted neuronal reorganization in the peri-infarct area and neurological function recovery following experimental cerebral ischemia (Beck et al., 2002).

Neurogenesis plays an important role in producing a full recovery of the damaged brain after stroke (Xin et al., 2017; Santopolo et al., 2020; Rahman et al., 2021). It is known that neural stem cells (NSCs) exist in the subventricular zone of the lateral ventricle and the dentate gyrus of the hippocampus and can differentiate into cells including neurons, astrocytes, and oligodendrocytes (Alvarez-Buylla et al., 2002; Arvidsson et al., 2002; Taupin and Gage, 2002). NSCs can be activated by diverse stimuli such as hypoxia (Vecera et al., 2020; Gengatharan et al., 2021). Recent study showed that Sema3A plays a pivotal role in promoting the differentiation of NSCs into neural cells in the injured cortical tissue (Figure 1A). In-depth study has found that the cross-talk between Sema3A and Wnt/β-catenin pathways participates in the regeneration process (Figure 1B; Xu et al., 2018). Another study indicated that Sema3A/NRP1 signaling is essential for cell differentiation into various essential cell types at defined target sites (Schwarz et al., 2009).

Glial cells were originally described as structural support in maintaining biological integrity. Accumulating evidence shows that glial cells act as a double-edged sword in the pathophysiology processes of various diseases including stroke (Abe et al., 2020). In the acute stage of ischemic stroke, glial cells activation could remove metabolic waste and produce anti-inflammatory cytokines and growth factors (Wanrooy et al., 2021). In the chronic stage of stroke, axonal regeneration is related to better prognosis. Activation of glial cells and glial scar formation create major inhibitory environments for axonal outgrowth in the peri-infarct area (Qin et al., 2019; Zhu et al., 2021). Astrocytes are involved in various pathophysiological processes in central nervous system (CNS), including homeostasis maintenance, synapse formation, structural support, cerebral blood flow regulation and BBB formation (Jha et al., 2018). Sema3A/NRP signal pathway can activate glial cells to exert phagocytosis which induces neuron apoptosis and participates in glial scar formation in ischemic stroke (Kaneko et al., 2006; Hou et al., 2008; Hira et al., 2018). Further study showed that MCAO rats treated with Sema3A inhibitor showed a significant improvement in motor function compared with the vehicle-treated rats. In addition, activation of astrocytes was suppressed by Sema3A inhibitor treatment. These results indicate that inhibition of Sema3A in the peri-infarct area suppresses activated astrocytes (Figure 1A; Hira et al., 2018). The underlying mechanism of axonal outgrowth is related to axonal GSK-3β expression and astrocyte-derived exosomes with prostaglandin D2 synthase expression (Figure 1B). In addition, Increasing IL-1β, released by microglial cells in ischemia, induces microvascular injury through the release of Sema3A from adjacent neurons and it can be reversed by knockdown of Sema3A (Rivera et al., 2013).

Vascular permeability disruption occurs during cerebral ischemia resulting in neuronal damage and prolonged loss of brain functions (Hou et al., 2015; Bernardo-Castro et al., 2020). Endothelial cells were damaged firstly in the ischemic region, which resulted in vascular permeability of damaged BBB and caused severe inflammation (Krueger et al., 2015; Ko et al., 2020). Sema3A acts as a potent inducer of vascular permeability *via* activation of NRP1 (Figure 1A; Acevedo et al., 2008). The expressions of Sema3A and NRP1 in endothelial cells after OGD treatment were up-regulated (Yang et al., 2019). However, vascular endothelial cell death was not apparent, which was associated with the increased generation of vascular endothelial growth factor (VEGF) after ischemia. VEGF/NRP signals promote angiogenesis in endothelial cells (Beck et al., 2002). As we know, NRP1 is a common receptor for the Sema3A and VEGF. The observations suggest that vascular NRP1 preferentially confers VEGF_164_ signals, while axonal NRP1 preferentially transmits Sema3A signals (Vieira et al., 2007). Hou et al. (2015) revealed that Sema3A bound to the NRP2/VEGFR1 receptor complex caused disorganization of F-actin stress fiber bundles and increased endothelial monolayer permeability, which contributes to ischemic brain damage (Figure 1B). VE (vascular endothelial)-cadherin expression is crucial for vascular permeability (Gavard, 2009; Treps and Gavard, 2017). Le Guelte et al. (2012) reported that Sema3A inhibits the serine protein phosphatase 2A (PP2A) activity and disrupts PP2A interaction with VE-cadherin, increasing vascular permeability (Figure 1B). Studies have shown that endothelial cells actively participate in synaptic plasticity in specific functional domains of brain to control some functions such as neurogenesis (Giacobini et al., 2014). Wu et al. (2019) found that Sema3A inhibited VSMC proliferation and migration by increasing the NRP1-plexinA1 complex and decreasing the NRP1- platelet-derived growth factors receptor (PDGFR)-β complex, thus inhibiting phosphorylation of PDGFR-β.

Pericytes are tightly connected to endothelial cells and distributed at discontinuous intervals in vascular basement membrane to maintain local microvessel characteristics (Hess et al., 2019). In CNS, pericytes contribute to the formation of the blood-brain barrier, and act as sensors of hypoxia and mediate precise responses to protect the vulnerable neurons (Dore-Duffy et al., 2005; Yang et al., 2017). Pericytes play a pivotal role in NVU injury in ischemic stroke (Duz et al., 2007). Casazza et al. (2011) found that Sema3A reduces the number of pericyte-coated vessels in tumor blood vessels, which correlated with endothelial cell survival. In ischemic stroke, pericytes respond to ischemia promptly and are involved in various pathological and repair processes. We conjectured that a connection between Sema3A and pericyte dysfunction which leads to the progression of vascular diseases such as stroke (Figure 1).

## Semaphorin 3B

Sema3B, another secreted member of the semaphorins, regulates axonal extension. Neuron dendritic structure in the motor cortex is associated with signal transmission of motor function and cell interaction. Ischemic stroke can damage dendritic structure, such as dendritic spine density, and induce motor deficits (Hartle et al., 2010; Huang et al., 2018). Dendritic spines contain different signaling molecules and machinery required for synaptic transmission and plasticity. Damaged dendritic spines cause cell-cell interaction dysfunction in NVU (Taylor et al., 2015). The L1 family Close Homolog of L1 (CHL1) is important for proper development of cortical networks (Pratte et al., 2003). Mohan et al. (2019) found that CHL1 was colocalized with Sema3B in pyramidal neurons and formed a complex with Sema3B receptor NRP2 and plexinA4. Treatment with Sema3B-Fc decreased spine density but did not induce spine retraction in CHL1-null neurons. This result indicated that CHL1 decreased spine density of cortical pyramidal neurons *via* stimulation by Sema3B (Mohan et al., 2019). CRMP not only plays a key role in axon guidance, but also regulates dendritic morphogenesis. A study showed that Sema3A signaling also regulated dendritic spine density *via* both CRMP1 and CRMP2 (Makihara et al., 2016). Another study found that dendritic spine density was decreased in cortical pyramidal neurons treated with semaphorin 3F (Sema3F) (Mohan et al., 2018). Thus, multiple semaphorin members can affect the integrity of neuron dendritic structure caused by ischemic stroke.

## Semaphorin 3E

Sema3E, an 85- to 90-kDa protein, was defined in tumor cells to play a role in angiogenesis (Hu and Zhu, 2018). At present, Sema3E and its receptors are thought to be closely related to stroke prognosis. Studies indicated that Sema3E and its receptor PlexinD1 inhibit cortical and striatal neurons development (Ding et al., 2011; Oh and Gu, 2013). In a rat transient middle cerebral artery occlusion model, Sema3E protein was increased in the penumbra area (Zhou et al., 2018a; Yu et al., 2021). Immunofluorescence study indicated that Sema3E staining is mainly colocalized with neurons and that the receptor PlexinD1 is expressed in endothelial cells in NVU. However, Inhibiting Sema3E signaling improves cerebral perfusion, functional outcome and survival after operation. On the other hand, Sema3E suppresses the migration capacity of pericytes toward endothelial cells, increases the vascular permeability, and damages NVU (Krueger et al., 2015; Zhou et al., 2018a). Mechanistically, Sema3E decreased dynamic delta-like 4 expression *via* inhibiting Ras-related C3 botulinum toxin substrate 1-induced c-Jun N-terminal kinase phosphorylation (Zhou et al., 2019).

In addition, the characteristics of atherosclerotic plaques are closely related to the development of ischemic stroke. However, upregulated Sema3E promotes plaque development by increasing macrophage migration and promoting macrophage retention and chronic inflammation (Wanschel et al., 2013). Therefore, Sema3E negatively regulates vascular permeability, inducing NVU damage, and inhibiting Sema3E signaling is a novel therapeutic strategy for ischemic stroke.

## Semaphorin 4D

**FIGURE 3:**
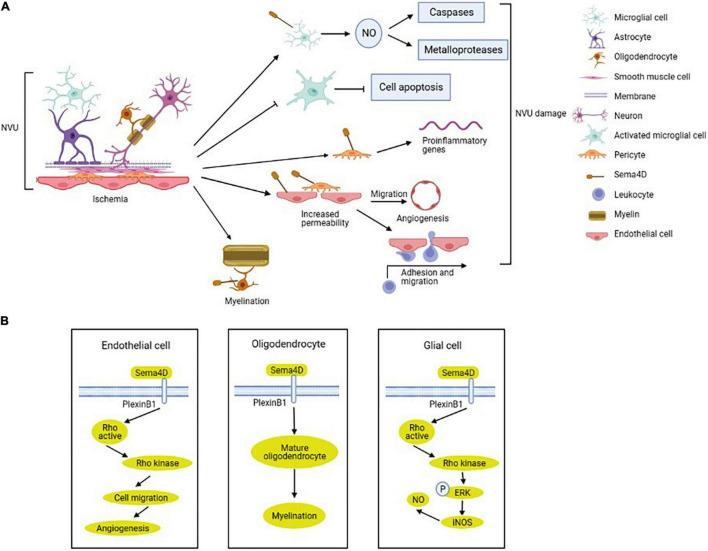
Regulation and mechanism of semaphorin 4D (Sema4D) in NVU after ischemic stroke. **(A)** The expression of Sema4D in NVU is up-regulated in ischemic stroke. Sema4D inhibits the NVU function by upregulating nitric oxide (NO) production in ameboid microglial cells, enhancing proliferation of activated-ramified microglial cells, promoting an inflammatory response in pericytes and endothelial cells, increasing vascular permeability and neovascularization. In addition, Sema4D increases neuronal myelination to protect the NVU. **(B)** Sema4D/Plexin-B1 promotes angiogenesis in endothelial cells *via* RhoA activation. Sema4D participate in the inhibition of axonal regeneration of oligodendrocytes, inhibiting remyelination. Sema4D/PlexinB1-RhoA-ERK signaling activate inducible nitric oxide synthase (iNOS), upregulating NO production in glial cells.

Sema4D, as a classic member of the semaphorin family and negative regulator of axon guidance, also regulates inflammation and angiogenesis by interacting with astrocytes, endothelial cells, and pericytes through its receptors plexins or CD72 (Figure 3A; Hu and Zhu, 2018). Sema4D was the first semaphorin that was determined to regulate inflammatory and immune response. Immune system functions rely on the interactions between leukocytes and endothelial cells *via* various adhesion molecules (Heemskerk et al., 2014). Furthermore, Sema4D works not only as a directional cue for endothelial cells migration, but also increases the expression of VEGF or angiopoietins to regulate angiogenesis (Conrotto et al., 2005). We could conclude that both the pathophysiological and neurovascular repair processes of ischemic stroke are strongly associated with the integrity of the NVU and that further investigations into Sema4D treatment targeted at the NVU could expand the therapies against deleterious outcomes following ischemic stroke.

Microglial-released proinflammatory and cytotoxic factors induced secondary brain damage after ischemia, and Sema4D inhibited LPS-induced microglial cells activation and migration (Toguchi et al., 2009). After ischemic stroke, the initial inflammatory response is mediated by the activation and recruitment of microglial cells, and inhibition of glial cells activation alleviates brain damage by ischemia (Li and Barres, 2018; Qin et al., 2019). The nitric oxide (NO) which is produced by the ischemia activated inducible nitric oxide synthase (iNOS), affecting cell survival by changing the functions of caspases and metalloproteases (Abdul-Muneer et al., 2013). Sema4D upregulates NO production by inducing IFN-β expression in microglial cells in the ischemic cortex (Sawano et al., 2019; Tsuchihashi et al., 2020). In ameboid microglial cells, L-arginine is metabolized by iNOS to synthesize NO through Sema4D-RhoA-MAPK/ERK signal (Figure 3B; Bijian et al., 2005; Okuno et al., 2010; Sawano et al., 2015). Decreased Sema4D expression enhances activated-ramified microglial cells proliferation which suppresses neuronal apoptosis in ischemic brain (Sawano et al., 2015). The study also indicated that Sema4D promoted cytotoxic activation of microglial cells in the peri-ischemic cortex (Sawano et al., 2015). Mechanistically, Sema4D/PlexinB1 signaling promotes an inflammatory response in pericytes and microglial cells and increases BBB permeability *via* regulating pericytes function after stroke (Zhou et al., 2018b).

Oligodendrocytes, the myelinating cells of the CNS, are involved in the recovery of neurological function by promoting the myelination of the damaged white matter (Poyhonen et al., 2019). A clinical study indicated that patients with SAO had significantly higher white matter hyperintensity compared with other stroke subtypes (Giese et al., 2020). Sema4D was expressed selectively by myelinating oligodendrocytes in the CNS white matter and upregulated after spinal cord lesion (Moreau-Fauvarque et al., 2003). Inhibition of Sema4D expression promotes oligodendrocytes recovery after cerebral ischemia/reperfusion injury in mice (Figure 3B; Wada et al., 2016).

Sema4D is expressed in endothelial cells and monocytes, and involved in endothelial-monocyte interaction, influencing migration and cytokines production (Luque et al., 2015). Under hypoxia, Sema4D expression was upregulated in microvascular endothelial cells. Overexpression of Sema4D significantly increases angiogenesis and inhibits neuron axon myelination (Zhang et al., 2014). Intraplaque neovascularization is important sites where leukocytes and macrophage infiltrate into atherosclerotic plaques and exacerbate atherosclerosis (Perrotta et al., 2021). Unstable atherosclerosis plaques are prone to rupture and induce thrombus formation, leading to ischemic stroke (Moreno, 2001). Inhibition expression of Sema4D reduces intimal neovascularization and plaque growth (Zhu et al., 2009; Yukawa et al., 2010). Mechanistically, Sema4D binding to plexinB1 on endothelial cells to guide endothelial cell migration induces intimal growth and angiogenesis (Figure 3B; Conrotto et al., 2005; Zhou et al., 2014). Furthermore, Sema4D also participates in endothelial-platelet interaction, increases endothelial cell permeability, and improves atherogenesis and thrombus formation (Conrotto et al., 2005; Zhu et al., 2007).

## Semaphorin 4B

Sema4B has been identified to be expressed in astrocytes of cortex and involved in the activation of astrocytes in brain injury. Astrocytes undergo dramatic changes in morphology, proliferation and gene expression after an ischemic insult (Choudhury and Ding, 2016). Sema4B significantly inhibited interleukin-4 production in response to various stimuli (Nakagawa et al., 2011). A study indicated that Interleukin-4 improves long-term neurological outcomes after stroke by reducing inflammation in the core and activated astrocytes in the penumbra (Xiong et al., 2011). Another study found that immunomodulation with IL-4 is a promising approach to promote long-term functional recovery after stroke through M2 phenotype induction in microglia/macrophages (Liu et al., 2016). Interaction between activated microglia and astrocytes plays an important role in the process of neuroinflammation after stroke (Liu et al., 2020). Mechanistically, damaged cortex activates astrocytes by phosphorylating the residues of Ser825 of Sema4B (Ben-Gigi et al., 2015). We can speculate that Sema4B serves as an astrocyte receptor may regulate astrogliosis after ischemic stroke. In-depth study of Sema4B may provide potential target for ischemic stroke treatment.

## Semaphorin 6A

Semaphorin 6A (Sema6A) regulates axon repulsion and attraction. Sema6A increases tumor angiogenesis *via* VEGF signaling in vascular endothelial cells (Segarra et al., 2012). Previous studies showed Sema6A was upregulated and improved functional recovery during the recovery phase in cortical ischemia (Kruger et al., 2006; Rogalewski et al., 2010). Good prognosis after stroke is partly associated with neurogenesis and changes in dendritic and synaptic morphology (Keyvani and Schallert, 2002; Santopolo et al., 2020). Like Sema3A, Sema6A also plays an important role in cortical neuronal networks rewiring after ischemia (Rogalewski et al., 2010). A study by Hatanaka et al. (2019) also indicated that Sema6A/plexinA2/A4 signaling regulates migration of superficial layer cortical neurons. Previous study showed that Sema6A mutant mice have corticospinal tract (CST) defect (Okada et al., 2019). Ischemic stroke is often accompanied by CST damage, however, whether Sema6A repairs the damaged CST has not been reported.

## Semaphorin 6B

Human Sema6B is highly expressed in human brain and regulates tumor growth (Correa et al., 2001). Accumulating evidence has been reported that peroxisome proliferator-activated receptor alpha activation modulates vascular integrity and function. It also modulates oxidative stress, blood-brain barrier dysfunction, and neuroinflammation to improve functional recovery from stroke (Boese et al., 2020). Proliferator-activated receptor alpha activation inhibits Sema6B expression and reverses Sema6B induced neuronal cell damage in the CNS (Collet et al., 2004; Inoue et al., 2016). It was reported that sema6B-plexinA4 signal promoted tumor angiogenesis by regulating VEGF-induced VEGFR-2 phosphorylation in endothelial cells (Kigel et al., 2011). We therefore speculate that proliferator-activated receptor alpha modulates the function of endothelial cells *via* sema6B-plexinA4 signal after ischemic stroke. However, the role of Sema6B in cerebrovascular disease has not been demonstrated using animal models.

## Semaphorin 7A

Sema7A, like other semaphorins, positively modulates axon guidance (Pasterkamp et al., 2003). Sema7A is expressed in a variety of neuronal cell types and in glial cells, and involved in multiple processes in the CNS, for example, acting as a potential immune and neuroregenerative target (Gutierrez-Franco et al., 2017; Jongbloets et al., 2017). Inflammatory responses are aroused by oxidative stress, necrotic cells, and impaired brain tissue (Amantea et al., 2009). Our previous studies indicated that Sema7A expression and its mediated inflammatory immune response in endothelial cells and monocytes participates in the development of atherosclerosis (Hu et al., 2018a,b). Subsequently, we conducted a study to investigate the association of serum Sema7A with atherothrombotic stroke and showed that elevated level of Sema7A is independently associated with atherothrombotic stroke (You et al., 2019). Oxidative stress induces injury of endothelial cells and neurons, and plays an important role in ischemic stroke. Oxidative stress significantly upregulates Sema7A and its receptor β1 integrin level, and activates inflammatory responses in endothelial cells (Song et al., 2021). Hypoxia and disruption of the BBB are the pathophysiological features of ischemic stroke, which significantly contribute to neuroinflammation and subsequent neurological disorders. During endothelial cell hypoxia, hypoxia-inducible factor-1α (HIF-1α) binds the Sema7A promoter hypoxia-responsive element to regulate inflammatory cell migration and leukocyte extravasation from the vascular space (Morote-Garcia et al., 2012).

## Other Members of Semaphorin Family

In addition to the members of semaphorin family described above, other semaphorins may be involved in the process of ischemic stroke as well although there was no report so far. Semaphorin 3C (Sema3C) and semaphorin 3D (Sema3D) play an important role in tumor development by regulating cell proliferation, migration, invasion, and angiogenesis processes (Valiulyte et al., 2019). FR-Sema3C is a point mutated form of Sema3C that is resistant to cleavage by furin like pro-protein convertases, which functions as an anti-angiogenic factor by inhibiting VEGF expression in endothelial cells (Toledano et al., 2016). In the developing cortex, interaction between matrix metalloproteinase-3 and Sema3C participated in the growth of axons and dendrites (Gonthier et al., 2007). Sema3D, like Sema3E, is capable of inhibiting endothelial cell motility, migration, and tube formation (Aghajanian et al., 2014; Taku et al., 2016). These evidence leads us to conjecture that Sema3C and Sema3D may be associated with vascular permeability and migration of neurons. Semaphorin 3F (Sema3F) modulates the morphology and function of synapses in the adult hippocampus. Mice lacking Sema3F are prone to seizures, suggesting that Sema3F is essential for the normal function of hippocampal circuits (Sahay et al., 2005). Semaphorin 3G (Sema3G) is secreted by the vascular system in the CNS and essential for the control of neural circuit stability and cognitive functions (Carulli et al., 2021). But there has been no direct evidence that Sema3F and Sema3G are associated with ischemic stroke.

Semaphorin 4A (Sema4A), like Sema4D, is immunomodulatory molecules in the immune cells. Sema4A binds to NRP-1 and promotes T cell activation and inflammation. In kidney ischemia reperfusion injury model, Sema4A alleviates inflammatory reaction by promoting the stability and function of regulatory T cells (Xu et al., 2021). Regulatory T cells are closely related to the pathogenesis of ischemic stroke. Semaphorin 4C (Sema4C) and semaphorin 4G (Sema4G), which are widely expressed in the developing nervous system, promote macrophage recruitment, angiogenesis and inflammatory reaction (Maier et al., 2011). Hence, semaphorin 4 may affect the development of brain ischemia reperfusion injury.

Semaphorin 5 has unique thrombospondin repeats as extracelluar domains. It’s well-known that semaphorin 5A (Sema5A) and its receptors play an important role in the invasion and metastasis of tumor cells by promoting angiogenesis (Sadanandam et al., 2010; Purohit et al., 2014). A study indicated that Sema5A was correlated with Th1 polarization, which increased the production of inflammatory cytokines (Lyu et al., 2015). A Th1-type response is neurotoxic and contributes to the poor outcome of stroke (Korhonen et al., 2015). Semaphorin 5B (Sema5B), as a repulsive guidance cue in the formation of the internal capsule, is expressed in the region of the cortex and subcortex (Lett et al., 2009). Sema5B also suppresses endothelial cell proliferation, migration and sprouting, and plays an important role in the regulation of neovascularization (Grundmann et al., 2013). The above evidence implies thatSema5A and Sema5B may contribute to the progression of vascular diseases such as stroke.

## Perspectives

Semaphorins are a large and diverse family of proteins involved in different physiological and pathological processes. Emerging evidence indicates that semaphorins not only regulate the shape and motility of neurons, but also relates with glial cell activity, blood-brain barrier (BBB) permeability, angiogenesis and inflammation/immune response in ischemic stroke. In this review, we summarized the role of semaphorins in NVU after stroke. Sema3A, a deeply understood member of the semaphorin family, mainly regulates the functions of neurons, glial cells, vascular system in the NVU. Sema4D and Sema7A signaling mainly participates in inflammatory response in pericytes and microglial cells after stroke. Like Sema3A, Sema3E and Sema4D can bind to their receptors directly on endothelial cells or affects VEGF expression to regulate neovascularization. The major roles of semaphorins in NVU after stroke are list in Table 1. Although there are limitations on the regulation the NVU function through a single semaphorin family member and its signaling pathway to improve functional recovery after ischemic stroke, coordination of the roles of different semaphorin members in the NVU and the successful clinical translational investigation could be potential approaches in prevention and treatment of ischemic stroke.

**TABLE 1:** Main roles of semaphorins in neurovascular unit (NVU) after stroke.

Semaphorins	Cells	Roles	References
Sema3A	Neuron	Neuron apoptosis, oxygen radical production, neurogenesis and cortical neuronal networks rewiring.	He et al., 2018; Xu et al., 2018; Yang et al., 2021; Zhao et al., 2021
	Glia	Astrocytes activation, glial scar formation	Hou et al., 2008
	Vasculature	Endothelial migration and death, VSMC proliferation and migration, angiogenesis, vascular permeability	Beck et al., 2002; Acevedo et al., 2008; Giacobini et al., 2014
Sema3B	Neuron	Neuron dendritic structure	Mohan et al., 2019
Sema3E	Vasculature	Pericytes ability, vascular permeability	Krueger et al., 2015; Zhou et al., 2018a
Sema4B	Glia	Activation of astrocytes.	Ben-Gigi et al., 2015
Sema4D	Glia	The activation and recruitment of microglial cells, inflammatory response, myelination of neuron,	Toguchi et al., 2009; Wada et al., 2016; Li and Barres, 2018
	Vasculature	Regulate endothelial-monocyte interaction, endothelial migration, neovascularization.	Conrotto et al., 2005; Zhang et al., 2014; Perrotta et al., 2021
Sema6A	Neuron	Cortical neuronal networks rewiring	Rogalewski et al., 2010
Sema6B	Vasculature	Vascular integrity	Collet et al., 2004; Inoue et al., 2016
Sema7A	Vasculature	Activates inflammatory responses in endothelial cells, vascular permeability	Morote-Garcia et al., 2012; Hu et al., 2018b; Song et al., 2021

## Author Contributions

HD and YX selected topics. HD wrote the review. LZ reviewed the manuscript and modified the content. All authors contributed to the article and approved the submitted version.

## Conflict of Interest

The authors declare that the research was conducted in the absence of any commercial or financial relationships that could be construed as a potential conflict of interest.

## Publisher’s Note

All claims expressed in this article are solely those of the authors and do not necessarily represent those of their affiliated organizations, or those of the publisher, the editors and the reviewers. Any product that may be evaluated in this article, or claim that may be made by its manufacturer, is not guaranteed or endorsed by the publisher.
